# Dietary total antioxidant capacity and its association with renal function and kidney stones: Results of a RaNCD cohort study

**DOI:** 10.1002/fsn3.2753

**Published:** 2022-04-19

**Authors:** Jalal Moludi, Arash Tandorost, Negin Kamari, Hadi Abdollahzad, Reza Pakzad, Farid Najafi, Yahya Pasdar

**Affiliations:** ^1^ 48464 Research Center for Environmental Determinants of Health (RCEDH) Kermanshah University of Medical Sciences Kermanshah Iran; ^2^ 48464 School of Nutritional Sciences and Food Technology Kermanshah University of Medical Sciences Kermanshah Iran; ^3^ 48432 Nutrition Research Center Faculty of Nutrition and Food Sciences Tabriz University of Medical Sciences Tabriz Iran; ^4^ 48443 Department of Epidemiology Faculty of Health Ilam University of Medical Sciences Ilam Iran

**Keywords:** chronic kidney disease, dietary total antioxidant capacity, kidney stones, RaNCD cohort, renal

## Abstract

There is evidence to support the hypothesis that dietary antioxidants have shown protective effects against chronic kidney disease (CKD). The purpose of this study was to determine the association between the dietary total antioxidant capacity (DTAC), renal function, and development of CKD and kidney stones in Ravansar Non‐Communicable Diseases (RaNCD) cohort study, Kermanshah, Iran. This cross‐sectional study was conducted using the recruitment baseline data of the RaNCD cohort study on 9,777 individuals aged 35–65 years. Food frequency questionnaire (FFQ) was performed to assess diet. DTAC scores were calculated using the ferric reducing antioxidant power (FRAP) of selected foods. Renal function was assessed by the estimated glomerular filtration rate (eGFR), blood urea nitrogen, and serum creatinine concentration. Prevalent CKD was based on an eGFR less than <60 ml/min per 1.73 m^2^. Incidence of kidney stones was also assessed by self‐reporting. Out of 9,777 participants, 1,747 subjects (eGFR: 18.50 ml/min per 1.73 m^2^; 95% confidence interval (CI): 17.72–19.30) had CKD. The mean DTAC score in this study was 0.24 ± 0.16 µmol TE/100 g (micromole of Trolox Equivalents). We showed a significant trend for eGFR across quartiles of DTAC, i.e., participants in the fourth quartile had a higher glomerular filtration rate (GFR) than those in the first one (DTAC _Q4 vs Q1_ = 82.20 versus 72.20 ml/min per 1.73 m^2^, *p* < .001). Another finding is that high DTAC scores were not associated with having kidney stones after adjusting for confounders. We revealed that higher DTAC scores have positive effects on the renal function. Interestingly, our findings showed that a high DTAC score had nonsignificant correlation with odds of kidney stones.

## Graphical abstract

To our knowledge, this is the first study designed to evaluate the relations between the dietary total antioxidant capacity (DTAC) and renal function and kidney stones in a large general population. Interestingly, we found an inverse and strong association between the renal function and DTAC, although we did not find a statistical significance for kidney stones and DTAC.

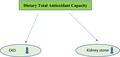

## 1 INTRODUCTION

Chronic kidney disease (CKD)—a situation with progressive decrease in renal function—is a major public health problem with an increasing incidence and prevalence (Mihai et al., 2018). A previous study revealed that the prevalence of CKD was 11.6% among Iranian adults (Kalantar‐Zadeh & Fouque, 2017; Levey & Coresh, 2012). Furthermore, the prevalence and incidence of kidney stones have continued to rise globally and appear to be a definite risk factor for CKD; therefore, patients with stones are suggested to undergo regular renal function tests. At present, it is expected that more than 24% of CKD cases in developed countries may be attributed to nutritional factors (Wang et al., 2008).

Studies on the quantity or quality of diet have demonstrated that adherence to Western dietary patterns is associated with increased risk of CKD (Rysz et al., 2017). The association between diet and the incidence of CKD may be mainly facilitated by insulin resistance and development of the hypertension, and diabetes (Spoto et al., 2016). Hence, healthy dietary patterns may have beneficial effects on the kidney function (Kurniawan et al., 2019). Lower plasma antioxidant levels and significantly reduced levels of antioxidant power have been shown in patients with CKD, which can result in an increase in diabetes complications (Saran et al., 2003). There is an existing hypothesis that a diet with high antioxidant capacity could be inversely related to the development of CKD (Psaltopoulou et al., 2011). The effectiveness of using various antioxidants, including vitamins, supplements, and some components of plants and fresh fruits, in attenuating the progression of kidney diseases and its complications has been suggested (Rysz et al., 2017). There is considerable literature on using single nutrients with antioxidant potential which have been shown to exert the protective effects on kidney function; however, the overall antioxidant capacity of the diet computing by a new tool called the dietary total antioxidant capacity (DTAC) defines synergistic actions of all antioxidants and is a practical predictor of diet–disease relationships (Nascimento‐Souza et al., 2018). The association between DTAC and many clinical outcomes including cardiovascular disease (CVD) and diabetes has been explored in prior studies (Mozaffari et al., 2018; Puchau et al., 2010). More recent evidence showed that the habitual intake of the dietary antioxidants is associated with a lower risk of CKD (Abbasi et al., 2019). However, population‐based cohort studies assessing the risk for CKD with DTAC are lacking. Taking advantage of the comprehensive medical record in the Ravansar Non‐Communicable Diseases (RaNCD) cohort study, we hypothesized that the higher antioxidant dietary intakes have potential for CKD management. Therefore, this study aimed to determine the dietary total antioxidant capacity (DATC) and its association with renal function and kidney stones in the RaNCD cohort study, Kermanshah, Iran.

## 2 METHODS

### 2.1 Study design and population

For this cross‐sectional study, we used the baseline data of Ravansar Non‐Communicable Diseases (RaNCD) cohort study that is one of the substudies of the national Prospective Epidemiological Research Studies in Iran (PERSIAN) (Poustchi et al., 2018). Ravansar is one of the western cities of Kermanshah Province with a population of about 50,000. For the RaNCD study, 10,000 participants aged 35–65 years were enrolled, covering approximately 75% of the eligible individual's residents in the area. The baseline phase of this study was completed during the years 2014–2017. The RaNCD study protocol has been published in detail (Pasdar et al., 2019). Data collection and whole measurements were conducted and assessed within the RaNCD cohort site. Invitation method had been done through a face‐to‐face appointment via visiting participants at their homes (Poustchi et al., 2018).

Subjects with a history of cancers, thyroid diseases, fatty liver disease, stroke, and end stage renal disease (ESRD) were excluded from the study due to the possibility of changes in eating habits. Subjects who take medications such as herbs, corticosteroids, and multivitamin supplements were not included in the analysis, since these supplements may skew the dietary outcomes on CKD.

### 2.2 Estimation of GFR

CKD (renal failure) is defined as kidney damage or GFR < 60 ml/min/1.73 (1.0 ml/s/1.73) present for more than 3 months. We calculated the estimated glomerular filtration rate (eGFR) from the Modification of Diet in Renal Disease (MDRD) equation, based on serum creatinine (Mihai et al., 2018), age, gender, and ethnicity by the following formula (Levey et al., 2006):

eGFR (ml/min per 1.73 m^2^) = 1.75 × Cr−1.154 × age−0.203 ×1.212 (if of African descent) × 0.742 (if female), where Cr is given in μmol/L and age in years, and the patients were categorized as CKD Stages 3–5 if the highest estimated GFR (eGFR) was <60 ml/min/1.73 m^2^.

### 2.3 Kidney stone

Incidence of kidney stones was assessed by self‐report, confirmed by medical record.

### 2.4 Dietary assessment

The habitual diet was assessed with a quantitative 137‐item food frequency questionnaire (FFQ). The trained interviewers asked participants to report how often, on average, they have consumed each food item daily, weekly, monthly, or yearly scale over last year. Updated dietary databases were used to calculate the quantity of nutrient intake (Pasdar et al., 2019).

### 2.5 Calculation of the DTAC

DTAC scores were calculated using the ferric reducing antioxidant power (FRAP) of selected foods. The ferric reducing antioxidant power (FRAP) assay is predicated on the reduction of a colorless Fe3+–TPTZ (2,4,6‐tripyridyl‐S‐triazine) complex into intense blue Fe2+–TPTZ, once it interacts with a possible antioxidant. At low cost, this method showed to be useful for the screening of antioxidant capacities and comparing efficiencies of various compounds (Leone et al., 2017).

### 2.6 Healthy eating index 2015

Healthy eating index (HEI) 2015 was calculated, based on the Krebs‐Smith et al. method (Levey et al., 2006). The HEI 2015 includes 13 items including total fruits, total protein foods, whole fruits, total vegetables, whole grains, seafood and plant proteins, greens and beans, dairy products, fatty acids, refined grains, sodium, added sugars, and saturated fats. For scoring the first six, the amount consumed is given a point from zero to 5 and the rest of the range of points is from zero to ten. Finally, the score from each item is added together and the final HEI score is between zero and 100.

### 2.7 Measurement of other variables

Type 2 diabetes is known as fasting blood glucose (FBG) levels equal to or greater than 126 mg/dl or those treated with hypoglycemic drugs. Patients with systolic and diastolic blood pressure equal to or greater than 140/90 mmHg, or those treated with blood pressure lowering medications, were considered as subjects with hypertension. Blood urea nitrogen (BUN) and serum creatinine (Mihai et al., 2018) concentration were measured by the enzymatic methods.

Standard physical activity questionnaire was used to assess the participants’ physical activity. The questionnaire consisted of 22 questions on the extent of individual activity during the circadian cycle. Answers were recorded within the questionnaire as hours or minutes per day. Finally, questionnaire information was extracted and used based on MET/hour per day (Hagströmer et al., 2006).

### 2.8 Statistical analysis

The association between variables was evaluated using univariate and multivariate logistic regression models. Variables with *p* < .05 in univariate analysis were entered into multivariate model. The fractional polynomial method was performed to quantitatively associate the effect of DTAC with an odds ratio of renal failure and kidney stone. In order to estimate the effect of DTAC on CKD and kidney stone, we entered confounding variables and then adjusted for diabetes, and high blood pressure, age, gender, smoking status, body mass index (BMI), education level, and physical activity. The effect of DTAC was then evaluated. The fractional polynomial is a regular polynomial alternative method that provides a flexible parameterization for continuous variables. All analyses were performed using Stata version 14.1 software (Stata Corp, College Station, TX, USA) with 95% confidence interval (CI).

## 3 RESULTS

Of the 10,065 individuals who participated in the RaNCD cohort, the status of CKD and other related indicators was filled for 9,777 (97.13%) participants and out of 9,777 participants, 1,747 subjects (eGFR: 18.50 ml/min; 95% CI: 17.72–19.30) had CKD. From them, 5,229 (53.41%) were female. The mean ± standard deviation (*SD*) of age for male and female was 47.6 ± 8.5 and 48.35 ± 8.83 years, respectively. As many as 2,691 (28.00) of participants had normal BMI and near 27% had low physical activity (<36 MET‐hours per day). The mean DTAC score in this study was 0.24 ± 0.16 µmol TE/100g and the DTAC score ranged from 0.01 (the most powerful oxidative diet) to 1.86 (the most powerful antioxidative diet) (Table 1).

**TABLE 1:** Mean ± standard deviation (*SD*) of dietary total antioxidant capacity (DTAC) in total and different quartiles based on the demographic factor

Variable		Total (*N* = 9,777)		Q1 (*N* = 2,444)	Q2 (*N* = 2,445)	Q3 (*N* = 2,444)	Q4 (*N* = 2,444)	*p* value
		9,777	0.236 ± 0.162	0.094 ± 0.024	0.161 ± 0.018	0.238 ± 0.028	0.449 ± 0.178	
Gender	Male	4,548	0.253 ± 0.161	0.100 ± 0.022	0.162 ± 0.019	0.239 ± 0.028	.445±.175	*<.001*
	Female	5,229	0.221 ± 0.160	0.092 ± 0.025	0.160 ± 0.019	0.238 ± 0.028	.455±.181	
Age group	35–45	4,587	0.248 ± 0.163	0.096 ± 0.023	0.161 ± 0.019	0.239 ± 0.028	.450±.172	*<.001*
	46–55	3,136	0.235 ± 0.162	0.096 ± 0.023	0.162 ± 0.019	0.238 ± 0.028	.454±.179	
	56–65	2,054	0.209 ± 0.155	0.090 ± 0.027	0.160 ± 0.019	0.236 ± 0.027	.441±.192	
Education level	Illiterate	2,431	0.193 ± 0.140	0.090 ± 0.026	0.159 ± 0.019	0.236 ± 0.027	.431±.175	*<.001*
	1–5 years	3,736	0.226 ± 0.157	0.096 ± 0.023	0.161 ± 0.018	0.238 ± 0.028	.449±.182	
	6–9 years	1,616	0.250 ± 0.161	0.100 ± 0.023	0.162 ± 0.019	0.239 ± 0.027	.450±.183	
	10.12years	1,233	0.270 ± 0.166	0.101 ± 0.021	0.162 ± 0.018	0.241 ± 0.028	.452 ± 0173	
	>13 years	761	0.334 ± 0.185	0.106 ± 0.016	0.167 ± 0.018	0.242 ± 0.027	.466±.170	
Place of residence	City	5,799	0.272 ± 0.176	0.097 ± 0.024	0.162 ± 0.019	0.240 ± 0.028	.459±.183	*<.001*
	Village	3,978	0.183 ± 0.119	0.093 ± 0.024	0.160 ± 0.019	0.235 ± 0.027	.412±.149	
Physical activity (MET‐hours per week)	24–36.5	2,993	0.242 ± 0.167	0.094 ± 0.024	0.162 ± 0.019	0.238 ± 0.028	.452±.182	*<.001*
	36.6–44.9	4,686	0.237 ± 0.166	0.094 ± 0.024	0.161 ± 0.018	0.239 ± 0.028	.454±.183	
	≥45	2,098	0.225 ± 0.141	0.097 ± 0.022	0.160 ± 0019	0.237 ± 0.027	.434±.154	
Smoking status	No	3,996	0.241 ± 0.166	0.094 ± 0.025	0.162 ± 0.019	0.237 ± 0.028	.455±.177	*<.001*
	Current	1,089	0.233 ± 0.145	0.096 ± 0.023	0.161 ± 0.019	0.241 ± 0.027	.419±.165	
	Former	829	0.240 ± 0.157	0.095 ± 0.023	0.163 ± 0.018	0.239 ± 0.027	.450±.176	
	Passive	3,752	0.229 ± 0.159	0.095 ± 0.024	0.160 ± 0.019	0.238 ± 0.028	.451±.177	
BMI (kg m^2^)	Under	170	0.210 ± 0.151	0.090 ± 0.026	0.154 ± 0.017	0.234 ± 0.031	.435±.143	*<.001*
	Normal	2,722	0.221 ± 0.150	0.093 ± 0.025	0.160 ± 0.018	0.238 ± 0.028	.436±.166	
	Overweight	4,200	0.239 ± 0.161	0.095 ± 0.024	0.162 ± 0.019	0.239 ± 0.029	.448±.176	
	Obese	2,577	0.247 ± 0.173	0.096 ± 0.023	0.161 ± 0.019	0.237 ± 0.027	.462±.189	
HEI	1st quintile	2,036	0.164 ± 0.113	0.090 ± 0.026	0.158 ± 0.018	0.235 ± 0.027	.422±.170	*<.001*
	2nd quintile	2,300	0.201 ± 0.123	0.095 ± 0.024	0.160 ± 0.019	0.236 ± 0.029	.414±.130	
	3rd quintile	1,541	0.227 ± 0.135	0.098 ± 0.22	0.160 ± 0.019	0.239 ± 0.026	.420±.134	
	4th quintile	2,139	0.266 ± 0.160	0.101 ± 0.020	0.164 ± 0.018	0.240 ± 0.028	.437±.168	
	5th quintile	1,761	0.334 ± 0.212	0.102 ± 0.022	0.165 ± 0.019	0.241 ± 0.029	.496±.211	
Kidney stone	No	7,986	0.234 ± 0.161	0.094 ± 0.024	0.160 ± 0.019	0.238 ± 0.028	.449±.178	*<.001*
	Yes	1,791	0.244 ± 0.163	0.096 ± 0.023	0.164 ± 0.019	0.240 ± 0.029	.451±.076	
Diabetes	No	8,885	0.235 ± 0.159	0.095 ± 0.024	0.161 ± 0.018	0.238 ± 0.028	.447±.173	*<.001*
	Yes	847	0.243 ± 0.184	0.090 ± 0.025	0.162 ± 0.019	0.241 ± 0.028	.479±.219	
Hypertension	No	8,232	0.238 ± 0.160	0.095 ± 0.024	0.161 ± 0.018	0.238 ± 0.028	.449±.173	*<.001*
	Yes	1,545	0.225 ± 0.166	0.092 ± 0.024	0.161 ± 0.019	0.238 ± 0.027	.455±.200	
GFR (ml/min per 1.73 m^2^)		9,717	76.72 ± 19.19	71.20 ± 18.54	76.66 ± 18.84	78.37 ± 19.07	82.80 ± 19.03	*<.001*
Urea (mg/dl)		9,717	13.56 ± 4.23	13.47 ± 4.18	13.44 ± 4.14	13.65 ± 4.53	13.67 ± 4.04	.*129*
Creatinine (mg/dl)		9,717	0.99 ± 0.003	1.01 ± 0.187	0.99 ± 0.243	0.99 ± 0.267	0.97 ± 0.170	*<.001*

Note

Data presented by mean ± *SD*.

Data were analyzed by one‐way and two‐way analysis of variance (ANOVA) test.

Table 1 also demonstrates general characteristics of subjects with CKD across quartiles of DTAC. There was a significant difference in the prevalence of diabetes (*p* < .001) and hypertension (*p* < .001) across quartiles of DTAC. No significant differences were detected in creatinine and urea concentrations across quartiles of DTAC. Mean levels of GFR, blood urea, and creatinine were 97.42 ml/min per 1.73 m^2^, 13.56 mg/dl, and 0.99 mg/dl, respectively. A significant difference was detected in creatinine and GFR across quartiles of DTAC.

Furthermore, the prevalence of kidney stones was 18.31% (95% confidence interval [CI], 17.56–19.10). The highest prevalence of kidney stone was observed in the Quartile (Q1) (the most powerful pro‐oxidative diet) (Table 1).

**FIGURE 1:**
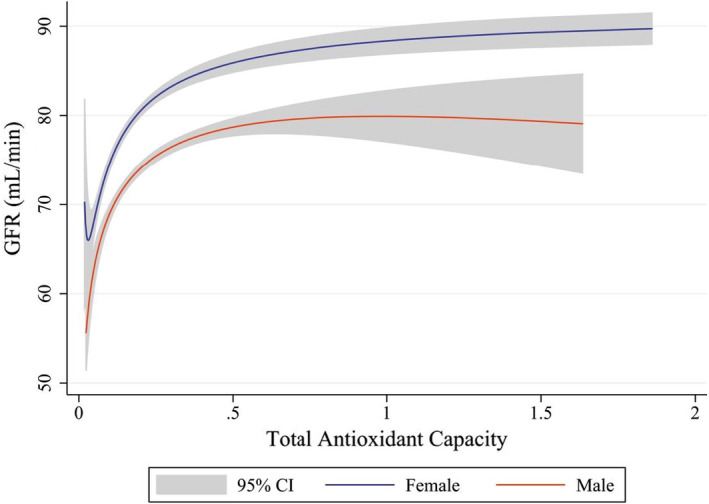
Association between glomerular filtration rate (GFR) with dietary total antioxidant capacity (DTAC) using fractional polynomial regression separated by gender

Figure 1 displays the mean of eGFR across level of DTAC. We could perceive a significant trend for eGFR across level of DTAC in men and women. Indeed, subjects with higher DTAC had higher eGFR, but the trend was similar in both sexes. Also, in female with a mean of eGFR 75–80, the trend of DTAC becomes smooth (plateau) but this pattern in male occurs in eGFR 85–90.

Overall, an inverse association was observed between DTAC and CKD, the odds of CKD in crude model in the fourth quartile were 0.42 (95% CI: 0.37–0.49) of the first quartile. In other words, in the fourth quartile of DTAC, the risk of CKD was 58% lower than the first quartile. After controlling the confounder factors, the odds ratio was increased compared to the crude model, but still the inverse association between DTAC and CKD was significant, so that in Model 3, adjusted for all important factors, the odds of CKD in Q4 (pro‐inflammatory) were 0.70 (95% CI: 0.57–0.87) of Q1 (anti‐inflammatory) (Table 2). It means that for people in the fourth quartile of DTAC, the risk of CKD was 30% lower than people in the first quartile of DTAC.

**TABLE 2:** Simple and adjusted odds ratio (OR) between dietary total antioxidant capacity (DTAC) with chronic kidney disease (CKD) and kidney stone using logistic regression

Variables	Models	DTAC quintile [OR (CI 95%)]				*P* trend
		Q1	Q2	Q3	Q4	
CKD	Crude	1	0.58 (0.50–0.66)	0.50 (0.43–0.57)	0.42 (0.37–0.49)	<0.001
	Model 1	1	0.69 (0.59–0.82)	0.64 (0.54–0.76)	0.64 (0.54–0.77)	<0.001
	Model 2	1	0.73 (0.61–0.88)	0.72 (0.59–0.87)	0.70 (0.56–0.86)	<0.001
	Model 3	1	0.73 (0.61–0.88)	0.72 (0.59–0.88)	0.70 (0.57–0.87)	<0.001
Kidney stone	Crude	1	1.09 (0.94–1.26)	1.18 (1.02–1.37)	1.18 (1.02–1.37)	0.013
	Model 1	1	1.03 (0.89–1.20)	1.11 (0.95–1.29)	1.08 (0.92–1.26)	0.243
	Model 2	1	1.02 (0.87–1.18)	1.08 (0.92–1.26)	1.03 (0.88–1.22)	0.579
	Model 3*	1	1.02 (0.87–1.18)	1.08 (0.92–1.26)	1.04 (0.88–1.22)	0.527

Note

Model 1: Adjusted for baseline age, gender, smoking status, body mass index (BMI), education level, and physical activity.

Model 2: Additionally, adjusted for healthy eating index (HEI).

Model 3*: Additionally, adjusted for diabetes and high blood pressure.

Also, a direct association was observed between DTAC and kidney stone, the odds of kidney stone in crude model in the fourth quartile were 1.18 (95% CI: 1.02–1.37) of the first one. After controlling the confounder factors, no association was observed between kidney stone and DTAC (Table 2).

**FIGURE 2:**
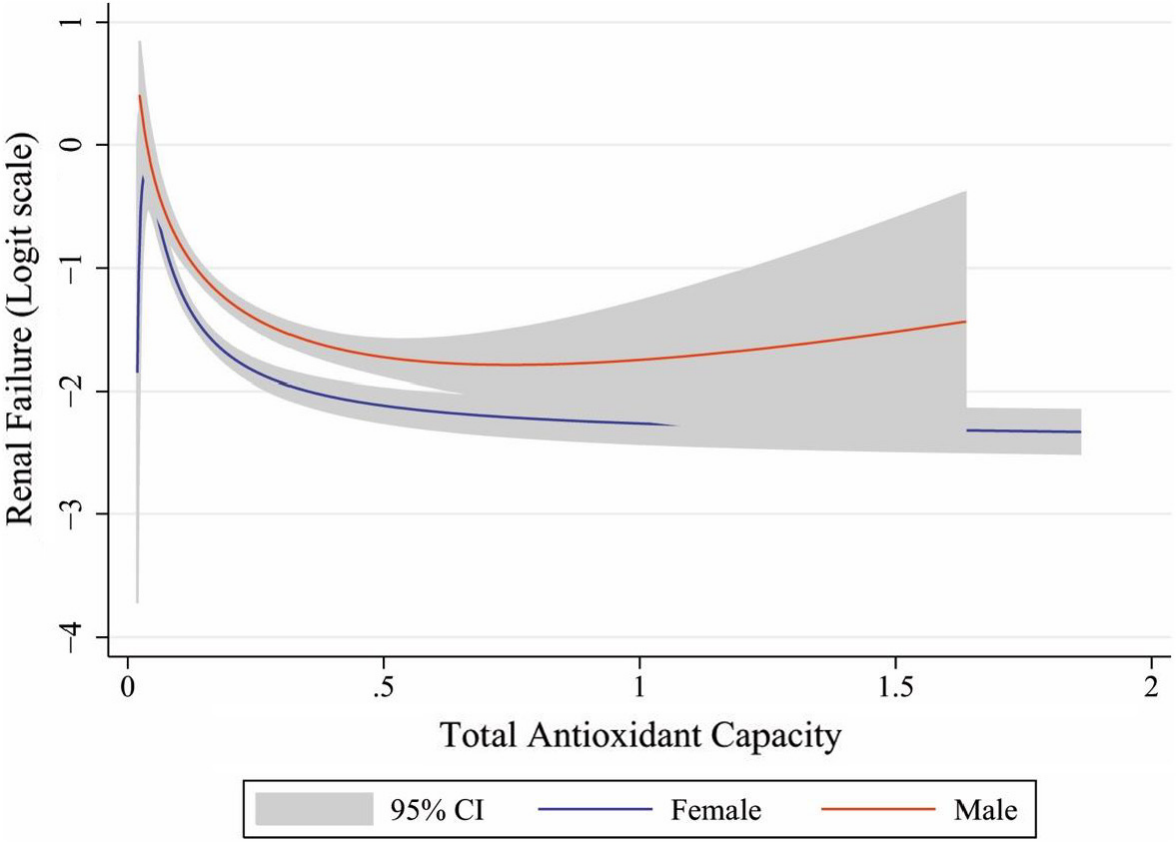
Odds of renal function with increase of dietary total antioxidant capacity (DTAC) separated by gender after adjusted confounders

Figure 2 shows the odds of renal failure across level of DTAC. The increases in the level of DTAC, the odds of renal failure decrease with a similar pattern both in men and women (*P* for trend = 0.001) but men have higher odds compared to women. Indeed, the results lead to the conclusion that men were more likely to get the renal failure disease than women.

**FIGURE 3:**
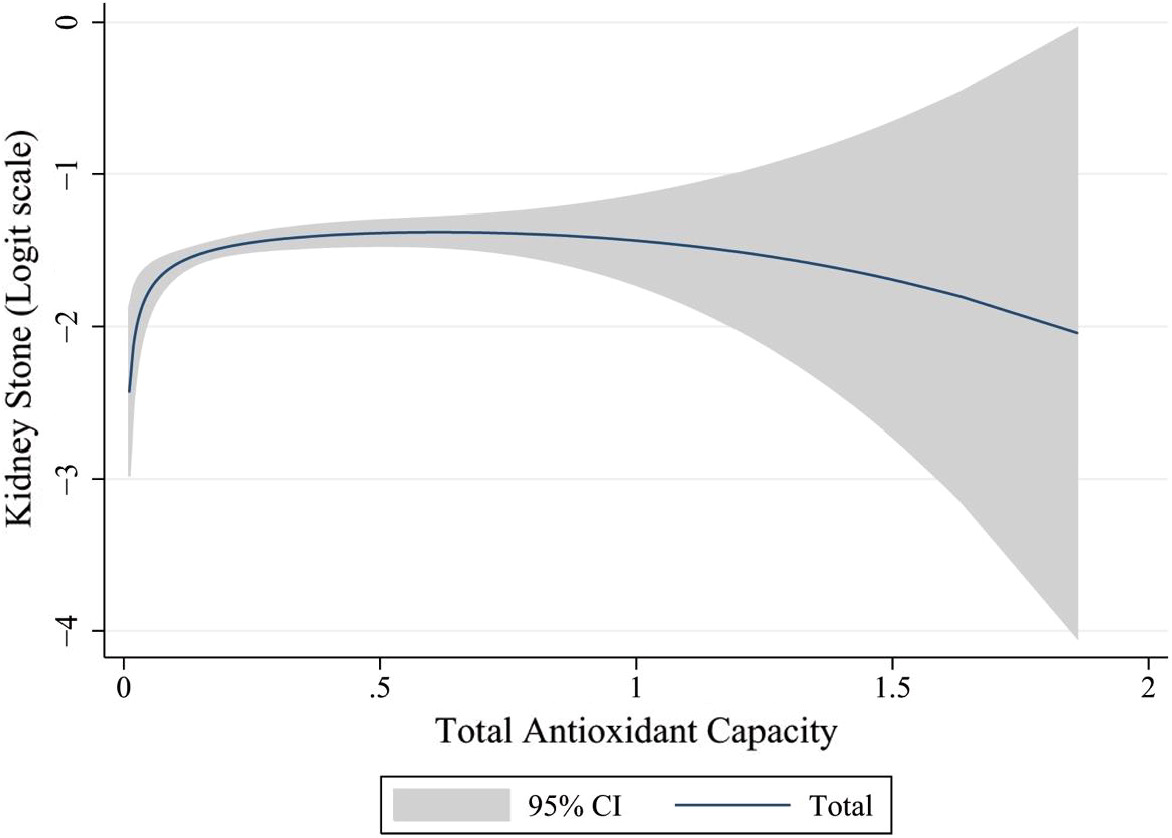
Odds of kidney stone with increase of dietary total antioxidant capacity (DTAC) separated by gender after adjusted confounders

Figure 3 shows the odds of kidney stone across level of DTAC. Although in a participant with the DTAC range 0–0.2, the odds of kidney stone increased (*P* for trend < 0.001), but for a participant with DTAC more than 0.2, the odds of kidney stone were plateau (*P* for trend = 0.468). The odds of kidney stone across level of DTAC were similar in male and female (Not shown in Figure 3).

As shown in Table 3; a positive association was observed between DTAC and eGFR, so that with one‐unit increase in DTAC, the mean of eGFR increased by about 19.10 (*p* < .001). Also, an inverse association was observed between DTAC and creatinine, so that with one‐unit increase in DTAC, the mean of creatinine decreased by about 0.06 (*p* < .001). There is no association between DTAC and urea.

**TABLE 3:** Association between dietary total antioxidant capacity (DTAC) with glomerular filtration rate (GFR), urea, and creatinine using linear regression

DTAC	Coefficient (95% CI)	*p*‐value
GFR (ml/min per 1.73 m^2^)	19.10 (16.71 to 21.48)	<.001
Blood urea (mg/dl)	0.10 (−0.42 to 0.63)	.693
Creatinine (mg/dl)	−0.06 (−0.09 to −0.04)	<.001

## 4 DISCUSSION

To our knowledge, this is the first study designed to evaluate the relations between DTAC with renal function and kidney stones in a large general population. Interestingly, we found an inverse and strong association between DTAC and renal function, however it was not significant for DTAC and kidney stones. Our study showed that patients in the highest quartiles of DTAC had an increased risk of being in the higher stage of CKD. The odds ratio of CKD in the fourth quartile is 0.70 times higher than in the first quartile. This finding is supported by prior studies (Mihai et al., 2018; Tajik et al., 2019). It has been suggested that poor dietary quality could lead to many metabolic dysfunctions including renal failure (Ahluwalia et al., 2013; Shivappa et al., 2014). A previous study has reported that diets high in whole grains, fruit or vegetables, and fish (anti‐inflammatory) are inversely related to the markers of inflammation including C‐reactive protein (CRP) and soluble intercellular adhesion molecule 1 (ICAM‐1), whereas a diet pattern rich in fats and processed meats (pro‐inflammatory) is directly related to CRP (Shivappa et al., 2018). Furthermore, it has been suggested that cytokine‐mediated inflammation is involved in the early stages of impaired kidney function during elderly, whereas cyclooxygenase‐mediated inflammation does not play a role at this stage (Nettleton et al., 2008). As a result, the anti‐inflammatory diet seems to be a possible preventive and therapeutic intervention in patients with CKD. Overall, former evidence was shown that diet quality has an important role in improving the CKD patient (Fernandes et al., 2018). The beneficial effects of antioxidants on health are documented. Higher intakes of fruit and vegetables, i.e., a diet rich in antioxidants, are related to a lower risk of several developing chronic diseases (Rule et al., 2011; Saucier et al., 2010). In a study from Taiwan that investigated 635 diabetic patients taking antioxidant‐rich foods, including fruit, vegetables, nuts, legumes, and whole grains, serum creatinine decreased and eGFR increased (Lin et al., 2010). In another study, patients with diabetes in the highest compared to the lowest tertiles of the modified alternate healthy eating index (HEI) score (high in plant‐based food) had 20% decreased risk of the occurrence of CKD (Gopinath et al., 2013). The Mediterranean diet (rich in antioxidants) has also been related to decreased risk of CKD occurrence (Nikniaz et al., 2018); these data, in line with our results, confirm that DTAC is associated with decreased risk of CKD incidence. In contrast, a case control study conducted by Abbasi et al. showed no significant association between DTAC and CKD in patients with type 2 diabetes (Pekgor et al., 2019).

Also, prior studies approved that people with kidney stones are prone to CKD in the future and it has been long been established that unhealthy dietary patterns could lead to kidney stones formation (Maddahi et al., 2017; Noori et al., 2014). In a previous study, it has been shown that adherence to an unhealthy dietary pattern, which was high in red meats and high fat diaries, is related to kidney stones formation (Maddahi et al., 2017). On the other hand, adherence to the Mediterranean dietary pattern (Leone et al., 2017) and DASH (Dietary Approaches to Stop Hypertension) diet (Noori et al., 2014), which are acting as anti‐inflammatory diets with high amounts of fruits, vegetables, and low‐fat dairy products, decreases in recurrent kidney stone formation have been seen (Rule et al., 2011; Saucier et al., 2010).

In this regard, our findings have shown that a high DTAC score presents a nonsignificant correlation with odds of kidney stones. Interestingly, the statistical analysis shows that a low score of DTAC is inversely related to kidney stones formation, but high scores of DTAC do not decrease the kidney stones formation risk.

Two previous epidemiologic studies have investigated the vitamin C relation with kidney stone in men. In a cohort study of men, 1,000 mg/day or more of total vitamin C intake was related to a 41% higher risk of stones, compared with 90 mg/day or less after adjusting for confounding factors (Kim et al., 2018). Another cohort study also reported a direct association between supplemental vitamin C intake and kidney stones in 23,355 Swedish men (Ramallal et al., 2015). As far as we know, no previous research has investigated the association of DTAC with kidney stones. Since the diet consists of different antioxidants that may perform synergistically and act with various mechanisms to prevent kidney stone formation, it seems more researches are needed to apply and test this relationship.

Statistical analysis in this study shows significant difference in the prevalence of diabetes (*p* < .001) across quartiles of DTAC. Aligned with our finding, other studies showed that pro‐inflammatory diet is associated with unfavorable glucose homeostasis (Phillips et al., 2018). In the cohort study of Okubo et al., DTAC has important protective effects on glucose tolerance, especially in older obese women (Iseki, 2008). A population‐based study emphasized on the beneficial effects of a diet rich in antioxidants on insulin resistance and risk of type 2 diabetes (Hsiao et al., 2015).

Also, our study shows significant difference in the prevalence of hypertension (*p* < .001) across quartiles of DTAC, which is in line with Bahadoran et al.’s study (Bahadoran et al., 2012 Dec). In contrast, no significant differences were detected in creatinine and urea concentrations across tertiles of the DTAC score, while a previous study reported that a dietary pattern rich in whole grains and fruit (anti‐inflammatory) was associated with lower urinary creatinine, whereas animal‐based food (pro‐inflammatory) intake was directly associated with higher creatinine across quintiles (Mazidi et al., 2018).

Thus, it is plausible that dietary antioxidants may attenuate CKD development and progression by compensating for imbalances in oxidative stress (Vivar Chevez et al., 2014).

One of the strengths of the present study is that this is the only study aimed to determine the association of DTAC with CKD among the Kurdish population in Iran with a large sample size. Second, this study could be on the high quality of data collection, population‐based study, and adjustment of all known confounding factors including age, gender, physical activity, and BMI. On the other hand, the use of DTAC rather than inflammatory markers to assess the impact of inflammation on clinical outcomes can help in direct measurement of the dietary impact on clinical outcomes through inflammation, and second reduction of the study costs. The calculation of DTAC through a cost‐effective and noninvasive method (FFQ) can evaluate the inflammatory properties of the diet. Likewise, the design of the current study was cross‐sectional, the causal relationship between DTAC and progression of CKD cannot be established, which is our study limitation and another study limitation is the self‐report for kidney stones.

## 5 CONCLUSION

In conclusion, it would appear that the highest quartiles of DTAC had decreased risk of being in the higher stage of CKD, improving renal function, and low occurrence of kidney stones in a large general population. Given the role of diet through antioxidant properties in the incidence of diseases such as CKD, it is recommended to consider the DTAC index in order to prevent, control, and treat the CKD, with more emphasis on dietary recommendations regarding the consumption of antioxidant diets. Further investigations are necessary to validate the kinds of conclusions that can be drawn from this study, especially about DTAC and kidney stones.

## CONFLICT OF INTEREST

The authors have declared no conflict of interest.

## ETHICAL APPROVAL

Informed written consents were obtained from all the candidates who were willing to participate, and they were ensured that they could withdraw from the study at any time they wished. The research was registered (No. 92472) at the Research and Technology Deputy and was approved by the Ethics Committee of the Kermanshah University of Medical Sciences.

## Data Availability

According to the RaNCD cohort protocols, datasets supporting the findings of this study are not publicly available. Data will be available from the corresponding author on reasonable request for researchers from other cohort centers.
